# Higher-order microbial interactions revealed by comparative metabolic modeling of synthetic communities with varying species composition

**DOI:** 10.1093/ismeco/ycaf142

**Published:** 2025-08-14

**Authors:** Dongyu Wang, Kristopher A Hunt, Britt Abrahamson, Zachary Flinkstrom, Xuanyu Tao, Ralph S Tanner, Kara B DeLeόn, Aifen Zhou, Jizhong Zhou, Michael J McInerney, Mari-Karoliina H Winkler, David A Stahl, Pieter Candry, Chongle Pan

**Affiliations:** School of Biological Sciences, University of Oklahoma, Norman, OK 73019, United States; Department of Biostatistic, Harvard T.H. Chan School of Public Health, Boston, MA 02115, United States; Department of Civil and Environmental Engineering, University of Washington, Seattle, WA 98195, United States; Department of Civil and Environmental Engineering, University of Washington, Seattle, WA 98195, United States; Department of Civil and Environmental Engineering, University of Washington, Seattle, WA 98195, United States; School of Biological Sciences, University of Oklahoma, Norman, OK 73019, United States; Institute for Environmental Genomics, University of Oklahoma, Norman, OK 73019, United States; School of Biological Sciences, University of Oklahoma, Norman, OK 73019, United States; School of Biological Sciences, University of Oklahoma, Norman, OK 73019, United States; Department of Chemical Engineering, Texas A&M University, College Station, TX 77843, United States; School of Biological Sciences, University of Oklahoma, Norman, OK 73019, United States; Institute for Environmental Genomics, University of Oklahoma, Norman, OK 73019, United States; School of Biological Sciences, University of Oklahoma, Norman, OK 73019, United States; Department of Civil and Environmental Engineering, University of Washington, Seattle, WA 98195, United States; Department of Civil and Environmental Engineering, University of Washington, Seattle, WA 98195, United States; Department of Civil and Environmental Engineering, University of Washington, Seattle, WA 98195, United States; Laboratory of Systems and Synthetic Biology, Wageningen University & Research, 6708 WE Wageningen, The Netherlands; School of Biological Sciences, University of Oklahoma, Norman, OK 73019, United States; School of Computer Science, University of Oklahoma, Norman, OK 73019, United States; Stephenson School of Biomedical Engineering, University of Oklahoma, Norman, OK 73019, United States

**Keywords:** high-order interaction, metabolic modeling, proteomics

## Abstract

Understanding how microbial interactions scale with community complexity is key to microbiome engineering and ecological theory. This study investigates emergent metabolic behaviors in controlled *in vitro* synthetic anaerobic communities of two, three, or four species: cellulolytic bacterium (*Ruminiclostridium cellulolyticum*), a hydrogenotrophic methanogen (*Methanospirillum hungatei*), an acetoclastic methanogen (*Methanosaeta concilii*), and a sulfate-reducing bacterium (*Desulfovibrio vulgaris*), representing core metabolic guilds in cellulose degradation and carbon conversion. We applied a systems biology framework combining proteogenomics, stoichiometric flux modeling, and SMETANA (Species Metabolic Coupling Analysis) to quantify syntrophic cooperation and competition across configurations. Cooperation peaked in tri-cultures and declined nonlinearly in more complex assemblies. Species roles shifted contextually. *Ruminiclostridium cellulolyticum* was the dominant donor, adjusting cellulase and hydrogenase expression by partner. *Methanosaeta concilii* became fully metabolite-dependent while enhancing methanogenesis. *Desulfovibrio vulgaris* improved syntrophic efficiency via redox and hydrogen turnover. In contrast, *Methanospirillum hungatei*’s metabolic centrality declined despite higher CH₄ output, suggesting interaction strength depends more on compatibility than richness. Reduced interactions in the four-species community stemmed from a single configuration and need further validation. This study moves beyond descriptive work by quantitatively resolving how metabolic networks rewire across defined communities. By characterizing context-dependent flux shifts at multiple layers, we provide a framework for interpreting and engineering stable, functionally interdependent microbial ecosystems.

## Introduction

Microbial transformation of cellulosic biomass into methane (CH₄) and carbon dioxide (CO₂) drives global biogeochemical cycles, shapes microbial communities in animal and human gastrointestinal tracts, and supports industrial applications. These processes influence carbon retention and release [1–3]. Within these communities, diverse species assemble into complex networks involving multiple levels of trophic interactions [4–7], resulting in cascades of cross-feeding. Cellulosic biomass is hydrolyzed into simpler compounds by primary degraders. Secondary consumers, such as methanogens and sulfate-reducing bacteria (SRBs), metabolize these intermediates into CH₄ and CO₂ [7, 8]. Interactions, both cooperative and competitive, within and among these groups shape microbial community structure, stability, and function, influencing ecosystem- and host-level processes [9–12].

The complexity and variability of natural microbial communities complicate the mechanistic understanding of biogeochemical processes. In particular, context-dependent cooperation and competition among microbial guilds hinder accurate prediction of community behavior [9, 13, 14]. While synthetic microbial communities (SynComs) provide tractable models, most prior research has focused on pairwise combinations or uncontrolled multispecies assemblies. As a result, the metabolic consequences of more complex community compositions remain poorly understood [15, 16]. Although omics studies enhance our understanding of microbial diversity and metabolic potential, they often fail to translate static taxonomic composition into predictive models of metabolic function [17–23].

In earlier work, we developed a synthetic microbial system composed of a cellulose hydrolyzer (*Ruminiclostridium cellulolyticum*), a hydrogenotrophic methanogen (*Methanospirillum hungatei*), an acetoclastic methanogen (*Methanosaeta concilii*), and a sulfate-reducing bacterium (*Desulfovibrio vulgaris*) [24]. These well-characterized anaerobes participate in cellulose degradation and carbon conversion in environments such as wetlands, the gut, and anaerobic digesters [25–28]. Their defined metabolic roles and genome-scale models made them ideal for constructing a minimal and mechanistically interpretable system. The design enabled complete combinatorial testing across bi-, tri-, and quad-species assemblies, allowing high-resolution analysis of interspecies metabolic interactions. This system revealed emergent synergies, such as increased CH₄ and CO₂ production in quad-cultures, as well as negative effects where increased species richness led to reduced cellulose degradation. These results highlight the nonlinear nature of microbial cooperation and competition, although the mechanisms driving these effects remain unclear [29–30].

**Figure 1:**
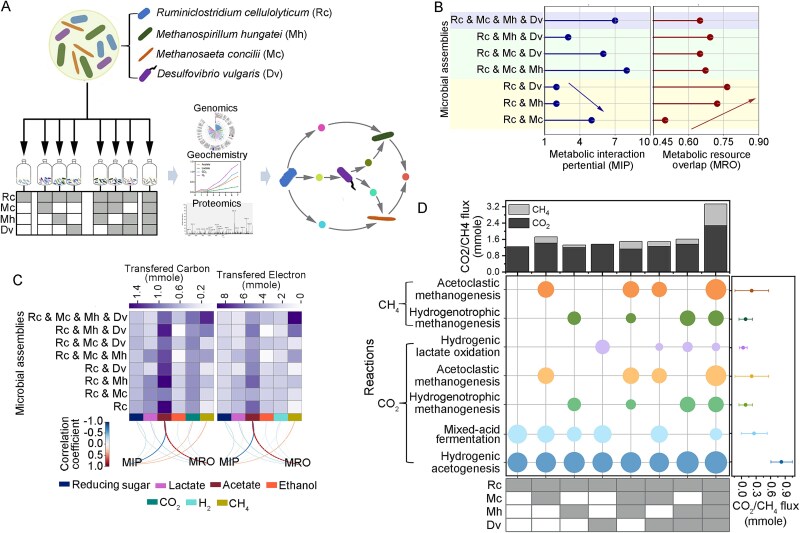
Experimental design and metabolic interactions in synthetic microbial communities. (A) Schematics of the combinatorial experimental setup. (B) Metabolic interaction potential (MIP) and metabolic resource overlap (MRO) across seven synthetic microbial communities. (C) Cumulative production of fermentation products, quantified by carbon and electron molarity, across different microbial assemblies. Heatmaps depict the transferred carbon and electron, with darker colors indicating higher transformation. Arcs illustrate the correlation between MRO and MIP for each microbial assembly, line thickness represents the strength of the correlation. (D) Fluxes of key metabolic reactions contributing to methane (CH₄) and carbon dioxide (CO₂) production. The circles represent seven days of reaction fluxes, and error bars indicate the range of fluxes observed in CH₄ and CO₂ production across eight microbial assemblies. Rc: *Ruminiclostridium cellulolyticum,* Mh: *Methanospirillum hungatei,* Mc: *Methanosaeta concilii*, Dv: *Desulfovibrio vulgaris.*

In this study, we propose that cooperation and competition are context-dependent, shaped by species-specific metabolic capacities and requirements in each configuration. Capturing this variability is essential for predicting microbial community succession. We combined the SMETANA (Species Metabolic Coupling Analysis) framework with proteogenomics and stoichiometric modeling to quantify the strength and direction of interspecies interactions [31, 32]. SMETANA provides two quantitative metrics: Metabolic Interaction Potential (MIP), which reflects cooperative metabolic exchange, and Metabolic Resource Overlap (MRO), which quantifies competition for shared substrates. These computational predictions were validated using metaproteomics and metabolic flux analysis, linking protein expression patterns to carbon partitioning from cellulose hydrolysis to CH₄ and CO₂ production (Fig. 1A). This work advances SynCom-based research by resolving how community composition alters interspecies cooperation and metabolic structure through higher-order interaction dynamics.

## Materials and Methods

### Genome-scale metabolic model building and community simulation

We used *Ruminiclostridium cellulolyticum* H10 (ATCC 35319), *Desulfovibrio vulgaris* Hildenborough (ATCC 29579), *M. hungatei* JF1 (txid323259), and *Methanosaeta concilii* (txid990316). Genomes and annotations were downloaded from NCBI. Genome-scale metabolic models (GEMs) were reconstructed using CarveMe, which generates draft models from genomic content [33]. Gap-filling was done using modified VM medium to ensure feasibility, and models were refined with BiGG and KEGG annotations. Community simulations were performed using SMETANA to estimate cross-feeding, metabolic dependencies, and interaction strengths [32]. Simulations used a virtual medium mimicking experimental condition. SMETANA ran with default settings, yielding three metrics: Metabolic Interaction Potential (MIP) indicates cooperative metabolite acquisition. MRO quantifies competition by shared metabolite import, and the SMETANA Score estimates syntrophic likelihood. Mathematical definitions are available in the original SMETANA publication [1].

### Experimental conditions and metabolite measurements

Experiments followed protocols from prior work [24]. Cultures were grown in 160 ml anaerobic bottles with 20 ml modified VM medium plus 10 g/L cellulose. *Ruminiclostridium cellulolyticum* and *D. vulgaris* were revived in VM and LS4D media at 34°C. *Methanospirillum hungatei* and *M. concilii* were pre-cultured in RST medium with H₂/CO₂ (80:20) and 50 mM acetate at 37°C. All strains were acclimated in VM at OD_600_ of 0.5 and inoculated at ~5 × 10^7^ cells per species. Cultures were incubated at 34°C, 200 rpm for 7 days under 3% H₂/97% N₂. Daily, 2 ml samples were centrifuged at 14 000 rpm for 15 min, supernatants were stored at −80°C. Headspace gas (5 ml) was collected in vacuum-sealed vials. Medium (2 ml) was replenished daily and pH reset to 7.3. Sulfate treatments received 2 ml of 100 mM K₂SO₄ daily, controls received water. Metabolites (acetate, lactate, ethanol, formate, glucose, cellobiose) were measured by HPLC with refractive index detection. Gases (H₂, CH₄, CO₂) were analyzed by GC with thermal conductivity detection, pressure was digitally recorded. All assays were performed in technical duplicates using standard curves. Detailed metabolite data are provided in Table S1.

### Calculation of Gibbs free energy change

Gibbs free energy under biological conditions (ΔG′) was calculated as:


$$ \Delta{G}^{\prime }=\Delta{G}^{{}^{\circ}\prime }+ RT\ \ln\ Q $$

where Δ*G*°′ is standard Gibbs energy at pH 7 and 25°C, *R* = 8.314 J/mol·K, *T* = 298.15 K, and *Q* is the reaction quotient from metabolite concentrations or gas pressures.

### Protein identification and quantification

Samples collected postincubation were processed using established protocols [34, 35]. Pellets were washed with nanopure water and lysed in buffer (10 mM Tris–HCl, 1% SDS, 0.1 M DTT) at 60°C for 1 h. Proteins were precipitated with trichloroacetic acid overnight at 4°C, washed with cold acetone, and resuspended in guanidine buffer. Concentrations were quantified via the Bicinchoninic Acid assay [36]. 20 mg protein samples were processed using filter-aided sample preparation, digested with Trypsin/LysC, and labeled using the TMTpro™ 16plex set. Peptides were fractionated into 46 groups and consolidated into 28 super-fractions. Each was further separated via UHPLC and analyzed on an Orbitrap Eclipse Tribrid MS with multi-notch MS3. MS1 scans were performed at 120000 resolution (375–1500 m/z). MS2 used CID (NCE 35) and ion trap detection. Synchronous precursor selection allowed HCD (NCE 65) and MS3 detection at 50000 resolution (100–500 m/z).

Spectra were searched using Proteome Discoverer v3.1 with SEQUEST against a custom four-species database. Peptides were filtered to 1% FDR [37, 38]. Protein groups were excluded from downstream analysis. Abundances were calculated from TMT intensity, normalized to dataset totals, and adjusted relative to each species’ total proteome abundance [39–41].

### Construction of the stoichiometric model

A set of overall metabolic reactions per species was constructed and fitted to observed metabolite levels (Figs S1 and S2). Adenosine Triphosphate (ATP) yields were sourced from KEGG, MetaCyc, and prior literature [42–45]. The model included pathways for cellulose degradation, hydrogenotrophic and acetoclastic methanogenesis, sulfate reduction, and fermentation. Protein abundances from metaproteomics were used to estimate pathway activity, assuming a positive correlation with reaction rates and scaled by ATP yield. This provided a semi-quantitative estimate of species-specific energy contributions. Deviations between model and measurements were attributed to biological variation or analytical limits. Code and data are available at: https://github.com/thepanlab/ProStoichiometric.

### Statistical analysis

Metabolite data were analyzed using Student’s *t*-test. Protein abundance differences were analyzed using the DESeq R package [46], with *q*-values calculated by the Benjamini–Hochberg method [46]. Proteins with *q* < 0.05 were considered significant.

## Results

### Microbial complexity enhances cooperation and methane output

Different combinations of *R. cellulolyticum*, *M. hungatei*, *M. concilii*, and *D. vulgaris*, including one mono-culture, three bi-cultures, three tri-cultures, and one quad-culture, were co-cultured in a medium with cellulose as the sole carbon source (Fig. 1A). These synthetic communities were analyzed using the SMETANA framework to compute MIP and MRO as proxies for cooperative metabolism and resource competition, respectively (Fig. 1B) [32].

Among bi-cultures, *R. cellulolyticum* with *M. concilii* had the highest MIP, indicating strong cooperation. In contrast, combinations with *M. hungatei* or *D. vulgaris* showed higher MRO, reflecting greater competition (Fig. 1B). Although these microbes utilize different primary substrates (cellulose, hydrogen, lactate), SMETANA inferred competition for shared imports like ammonium, phosphate, glycerol, and ferric iron. These micronutrients are essential for biosynthesis, so MRO values reflect auxiliary, not primary substrate, competition.

Adding a third species to form tri-cultures significantly altered MIP-MRO dynamics. The *R. cellulolyticum*–*M. concilii*–*M. hungatei* tri-culture showed 60% and 300% MIP increases over respective bi-cultures, and a 45% MRO decrease. Similarly, the *R. cellulolyticum*–*M. concilii*–*D. vulgaris* tri-culture increased MIP by 20% and 200% and reduced MRO by 48%. The *R. cellulolyticum*–*M. hungatei*–*D. vulgaris* tri-culture showed a 50% MIP increase and 53% MRO decrease. These results suggest that tri-cultures enhance cooperation and reduce competition.

In the quad-culture, MIP dropped by 22% compared to cumulative bi-cultures, but MRO declined by 76% (Fig. 1B), suggesting that while cooperation may diminish slightly, reduced competition stabilizes metabolism in the full community.

To assess functional outcomes, carbon and electron transfers were measured (Fig. 1C). The *R. cellulolyticum*–*M. hungatei* bi-culture transferred 0.42 ± 0.007 mmol carbon and 3.33 ± 0.06 mmol electrons to CH₄. Adding *D. vulgaris* increased both values by 112%. In the quad-culture, carbon and electron transfer reached 1.37 ± 0.03 mmol and 10.99 ± 0.21 mmol, a 226% increase over the corresponding tri-culture and combined bi-cultures. These findings suggest *D. vulgaris* functions as a syntrophic lactate oxidizer that facilitates hydrogen transfer and supports methanogenesis. Acetate production was negatively correlated with MIP (*r* = −0.69, *P* = .002) and positively with MRO (*r* = 0.76, *P* = .005), indicating that acetate accumulates under competition and declines with cooperation.

Metaproteomics identified 1296 proteins across all eight communities using species-specific peptides (Table S2). A refined stoichiometric model incorporated six key reactions [24]: lactate fermentation, hydrogenic acetogenesis, mixed-acid fermentation, hydrogenic lactate oxidation, hydrogenotrophic methanogenesis, and acetoclastic methanogenesis (Fig. S1), with ATP yields incorporated. Reaction contributions were fitted to cumulative measurements of lactate, acetate, ethanol, H₂, CO₂, CH₄, and biomass. Model performance was high (*R*^2^ > 0.9, NRMSE = 0.084) (Fig. S2), validating calculated carbon and energy fluxes. Flux analysis identified acetoclastic methanogenesis as the primary CH₄ and CO₂ source, especially in multi-species cultures. This pathway was dominant for methane production and energy conservation. Total carbon and electron fluxes to CH₄ and CO₂ in tri- and quad-cultures exceeded the sum of bi-cultures, revealing synergistic, non-additive interactions. These results, consistent with previous findings [24], emphasize acetoclastic methanogenesis as a central driver of emergent cooperation in complex communities (Fig. 1D).

### Community context shapes cellulose degradation and fermentation in *R. cellulolyticum*

**Figure 2:**
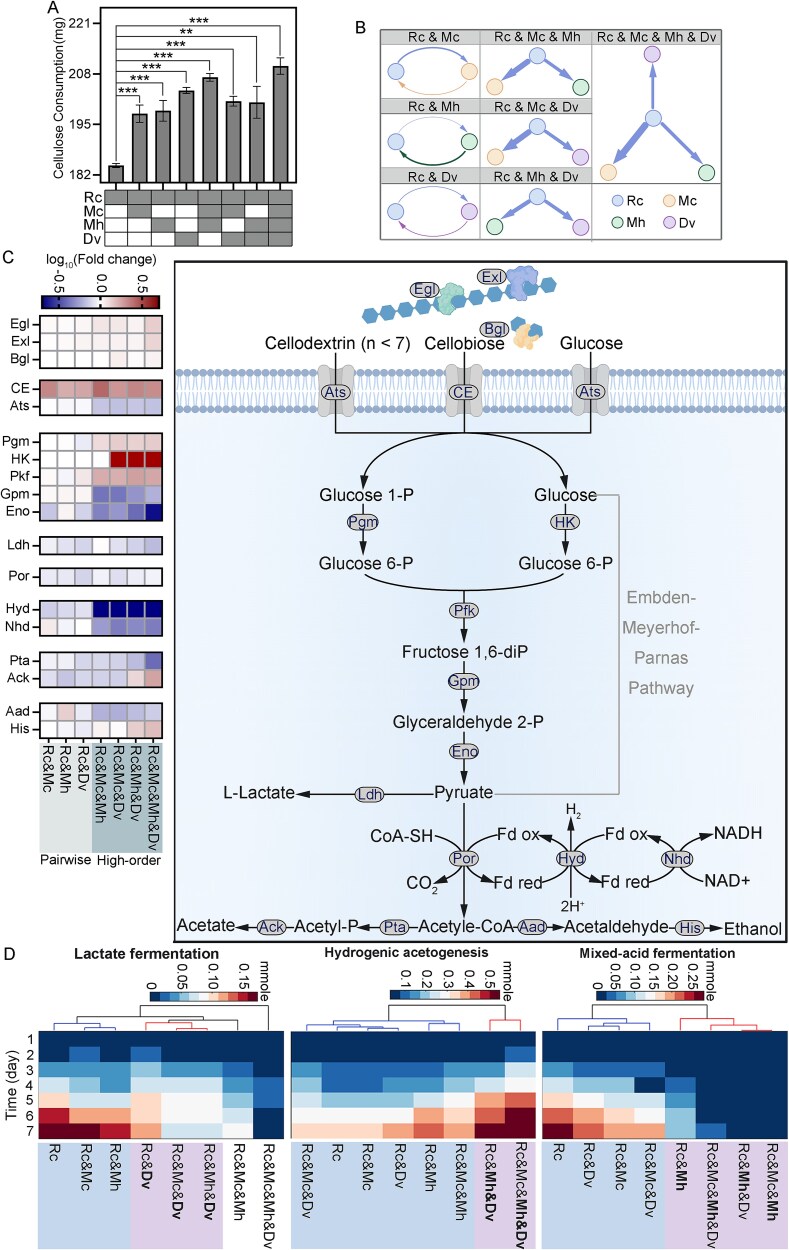
Metabolic activities of *R. cellulolyticum* in synthetic communities. (A) Comparison of cellulose consumption by *R. cellulolyticum* among different cultures. Significant differences with *P*-values determined using Student’s *t*-test, and adjusted for the false discovery rate, are marked by ^*^ for *P*-value < .05, ^**^ for *P*-value < .01, and ^***^ for *P*-value < .001. The error bars are defined as standard deviation. (B) Cross-feeding interactions between *R. cellulolyticum* and other species in all the synthetic communities. (C) Protein expression profile of the major carbon metabolism pathways in *R. cellulolyticum*. (D) Fluxes of the key reactions in *R. cellulolyticum* modeled in all cultures. Rc: *Ruminiclostridium cellulolyticum,* Mh: *Methanospirillum hungatei,* Mc: *Methanosaeta concilii*, Dv: *Desulfovibrio vulgaris.* Egl: β-endoglucanase, Exl: Β-exoglucanase, Bgl: β- glucosidase, CE: cellobie 2-epimerase, Ats: ABC transporter, pgm: phosphoglucomutase, HK: hexokinase, Pfk: 6-phosphofructokinase, Gpm: 2,3-bisphosphoglycerate-dependent phosphoglycerate mutase, Eno: Enolase, Ldh: lactate dehydrogenase, Por: pyruvate:ferredoxin oxidoreductase, Hyd: periplasmic [NiFeSe] hydrogenase, Nhd: NADH-fd reductase, Pta: phosphate acetyltransferase, Ack: acetate kinase, Aad: acetaldehyde dehydrogenase, His: alcohol dehydrogenase.

Cellulose degradation by *R. cellulolyticum* increased in the presence of other species (Fig. 2A), with cocultures showing higher cellulase protein abundance than the mono-culture, as revealed by metaproteomics (Fig. 2C). However, the enhancement showed weak correlations with MIP (*r* = 0.30, *P* = .51) and MRO (*r* = 0.35, *P* = .40), suggesting that predicted metabolic interactions alone cannot explain cellulolytic activity. MIP and MRO capture community-level exchange potential and competition but do not account for species-specific regulation, spatial interactions, or local conditions that influence cellulase expression.

Cellulose degradation efficiency varied with species composition. Over 7 days, the *R. cellulolyticum* mono-culture degraded 184.4 ± 0.5 mg of cellulose. Coculture with *D. vulgaris* increased degradation by 10.5% (*P* = .002), but adding *M. concilii* reduced it by 3% (*P* = .03), and *M. hungatei* had no significant effect (*P* = .2). Including both methanogens with *R. cellulolyticum* and *D. vulgaris* in a quad-culture led to a 3% increase over the bi-culture (*P* = .008) and a 5% increase compared to the tri-cultures with *M. concilii* (*P* = .003) or *M. hungatei* (*P* = .02). These results suggest that *D. vulgaris* enhances degradation, while methanogens have context-dependent effects.

SMETANA-based simulations revealed metabolic interdependencies in these communities (Tables 1 and 2, Fig. 2B) [31]. The SMETANA score quantifies essential metabolite exchange between species pairs. Higher scores indicate stronger dependence, while lower scores suggest greater autonomy. Each species may act as a donor or a receiver. In the *R. cellulolyticum*–*D. vulgaris* bi-culture, mutualism was evident; the score was 2.12 when *R. cellulolyticum* was the donor and 2.46 as the receiver. This likely reflects facultative syntrophy, where *R. cellulolyticum* supplies lactate and hydrogen to *D. vulgaris*, and receives ammonium, sulfur compounds, and small organics in return. In contrast, in bi-cultures with *M. concilii* or *M. hungatei*, *R. cellulolyticum* had high donor scores (4.60 and 4.85), but receiver scores dropped by 47.8% and 83.1%, respectively. This asymmetry suggests *R. cellulolyticum* contributes more than it gains from methanogens, unlike its mutualistic relationship with *D. vulgaris*. In tri- and quad-cultures, *R. cellulolyticum* remained the dominant donor, reinforcing its ecological role in cellulose breakdown and provision of intermediates to syntrophic partners.

**Table 1:** Summary of metabolic interactions, stoichiometric modeling, and proteomic analyses within tri-cultures.

Microbial assembly	Species pair		SMETANA	Stoichiometric model			Proteomics	
	Donor	Receiver	Fold change	Metabolite	Reaction	Fold change (*P-*value)	Protein name	Fold change (*P-adj*)
**Rc & Mc** & Mh			1.55 ▲	Acetate	Hydrogenic acetogenesis	1.08 (^*^) ▲	Acetate kinase	ns
	Rc	MC			Mixed-acid fermentation	> 100 (^***^) ▼	Aldehyde-alcohol dehydrogenase	1.89 (^***^) ▼
**Rc & Mc** & Dv			1.55 ▲		Hydrogenic acetogenesis	1.13 (^***^) ▼	Acetate kinase	ns
					Mixed-acid fermentation	ns	Aldehyde-alcohol dehydrogenase	1.79 (^*^) ▼
**Rc & Mh** & Mc			1.13 ▼	H_2_, CO_2_	Hydrogenic acetogenesis	1.10 (^**^) ▼	Pyruvate flavodoxin/ferredoxin oxidoreductase	1.49 (^**^) ▼
	Rc	Mh			Mixed-acid fermentation	> 100 (^***^) ▼	Aldehyde-alcohol dehydrogenase	1.89 (^***^) ▼
**Rc & Mh** & Dv			1.06 ▼	H_2_, CO_2_	Hydrogenic acetogenesis	1.26 (^**^) ▲	Pyruvate flavodoxin/ferredoxin oxidoreductase	1.56 (^*^) ▼
					Mixed-acid fermentation	> 100 (^***^) ▼	Aldehyde-alcohol dehydrogenase	2.28 (^*^) ▼
**Rc & Dv** & Mc			1.34 ▲	Lactate	Lactate fermentation	1.56 (^*^) ▼	Lactate dehydrogenase	1.16 (^*^) ▲
**Rc & Dv** & Mh	Rc	Dv	2.02 ▲			1.53 (^*^) ▼		ns
**Rc & Mc** & Mh			>100 ▼	Acetate	Acetoclastic methanogenesis	ns	Methyl-coenzyme M reductase	2.80 (^***^) ▲
**Rc & Mc** & Dv	Mc	Rc	>100 ▼			1.38 (^**^) ▼		2.08 (^***^) ▲
**Rc & Mh** & Mc			>100 ▼	H_2_, CO_2_	Hydrogenotrophic methanogenesis	3.29 (^*^) ▼	Formylmethanofuran dehydrogenase	> 30 (^***^) ▲
**Rc & Mh** & Dv	Mh	Rc	>100 ▼			1.81 (^*^) ▲	Formylmethanofuran dehydrogenase	> 30 (^***^) ▲
**Rc & Dv** & Mc	Dv	Rc	>100 ▼	Lactate	Hydrogenic lactate oxidation	9.30 (^***^) ▼	Phosphate acetyltransferase	1.36 (^*^) ▼
**Rc & Dv** & Mh			>100 ▼			2.83 (^***^) ▼		1.31 (^*^) ▼

▲, significant increase; ▼, significant decrease; -, no significant changes were observed.

^*^For *Padj* < .05, ^**^for *Padj* < .01, and ^***^for *Padj* < .001.

**Table 2:** Summary of metabolic interactions, stoichiometric modeling, and proteomic analyses within quad-culture.

Species Pair		SMETANA	Stoichiometric model			Proteomics	
Donor	Receiver	Fold change	Metabolite	Reaction	Fold change (*P*-value)	Protein name	Fold change (*P-adj*)
Rc	Mc	1.73 ▲	Acetate	Hydrogenic acetogenesis	1.53 (^**^) ▲	Acetate kinase	2.15 (^***^) ▲
				Mixed-acid fermentation	2.72 (^*^) ▼	Aldehyde-alcohol dehydrogenase	ns
Rc	Mh	1.20 ▼	H_2_, CO_2_	Hydrogenic acetogenesis	1.30 (^***^) ▲	Acetate kinase	ns
				Mixed-acid fermentation	1.47 (^*^) ▼	Aldehyde-alcohol dehydrogenase	14.2 (^***^) ▲
Rc	Dv	1.42 ▲	H_2_, CO_2_	Lactate fermentation	5.18 (^***^) ▼	Lactate dehydrogenase	1.18 (^*^) ▼
Mc	Rc	>100 ▼	Acetate	Acetoclastic methanogenesis	2.55 (^***^) ▲	Methyl-coenzyme M reductase	> 30 (^***^) ▲
Mh	Rc	>100 ▼	H_2_, CO_2_	Hydrogenotrophic methanogenesis	1.83 (^**^) ▲	Formylmethanofuran dehydrogenase	>30 (^***^) ▲
Dv	Rc	>100 ▼	Lactate	Hydrogenic lactate oxidation	9.97 (^***^) ▼	Phosphate acetyltransferase	> 30 (^***^) ▲
Mc	Mh	NA	CO_2_	Acetoclastic methanogenesis	2.53 (^***^) ▲	Methyl-coenzyme M reductase	2.98 (^***^) ▲
Mh	Mc	1.22 ▼	CO_2_	Hydrogenotrophic methanogenesis	6.01 (^***^) ▲	Formylmethanofuran dehydrogenase	ns
Dv	Mc	1.27 ▼	Acetate	Hydrogenic lactate oxidation	ns	Phosphate acetyltransferase	15.11 (^***^) ▲
Dv	Mh	NA	H_2_	Hydrogenic lactate oxidation	3.52 (^*^) ▼	Phosphate acetyltransferase	ns

▲, significant increase; ▼, significant decrease; -, no significant changes were observed.

^*^For *Padj* < .05, ^**^ for *Padj* < .01, and ^***^ for *Padj* < .001.

Metaproteomics revealed changes in *R. cellulolyticum*’s fermentation enzyme profiles across configurations (Fig. 2C). Compared to mono-culture, sugar-related ABC transporter (Ats) protein levels were similar in bi-cultures but decreased over 30-fold in tri- and quad-cultures (*q* < 0.0001), suggesting reduced substrate uptake in complex communities. Protein levels of NADH-fd reductase (Nhd), acetaldehyde dehydrogenase (Aad), and alcohol dehydrogenase (His) increased significantly in bi-cultures (fold change >30, *q* < 0.0001), but declined in tri- and quad-cultures (*q* < 0.0001), indicating community-driven restructuring of fermentation pathways. Hydrogenase (Hyd) protein abundance, linked to H₂ production, dropped by 1.37-fold in the *R. cellulolyticum*–*M. concilii* bi-culture and by over 30-fold in tri- and quad-cultures (*q* < 0.0001). This suggests active suppression of H₂ production to maintain low hydrogen levels favorable for syntrophy. In simpler cultures, *R. cellulolyticum* likely increases H₂ production to boost ATP yield. In more complex communities, efficient H₂-consuming partners relieve this burden, allowing metabolic resources to shift. This highlights cooperative adaptation and metabolic flexibility.

To quantify these transitions, we modeled flux through *R. cellulolyticum*’s key fermentation pathways—lactate fermentation, mixed-acid fermentation, and hydrogenic acetogenesis—across all community types (Fig. 2D). Lactate fermentation flux dropped significantly with *D. vulgaris*, declining by 88% in the quad-culture relative to mono-culture (*P* < .0001). Although exergonic (ΔG′ ≈ −32 kJ/mol) (Table 3), this flux likely decreased due to lactate consumption reducing its thermodynamic drive. Mixed-acid fermentation also declined in the presence of *M. hungatei* and was undetectable in the *R. cellulolyticum*–*M. hungatei*–*D. vulgaris* tri-culture. Despite being moderately exergonic (ΔG′ ≈ −26 kJ/mol), reduced activity may reflect a redirection of reducing equivalents toward more favorable reactions under syntrophy. In contrast, hydrogenic acetogenesis flux increased in the presence of other species. It rose by 58.8% in the tri-culture with *M. hungatei* and *D. vulgaris* (*P* = .0004), and by 61.8% in the quad-culture (*P* = .0009), relative to mono-culture. This pathway showed the greatest ΔG′ improvement, from −7.5 kJ/mol in mono-culture to nearly −29 kJ/mol in quad-culture, driven by H₂ removal. These findings show that *M. hungatei* and *D. vulgaris* promote a thermodynamically favorable shift from classical fermentation to hydrogenic acetogenesis, supporting cooperative energy flow and enhanced facultative syntrophy.

**Table 3:** Gibbs free energy (ΔG°, in kJ/mol) of key metabolic reactions across different microbial community assemblies.

Assemblies	Lactate fermentation	Hydrogenic acetogenesis	Mixed-acid fermentation	Hydrogenic lactate oxidation	Hydrogenic methanogenesis	Acetoclastic methanogenesis
Rc	−106.54	−76.74	−134.71	−24.02	−191.83	−105.06
Rc&Dv	−103.15	−75.54	−131.81	−22.89	−133.41	−44.69
Rc&Mc	−103.24	−76.49	−131.78	−23.62	−129.84	−40.83
Rc&Mc&Dv	−104.63	−74.89	−131.68	−22.80	−130.82	−41.36
Rc&Mc&Mh	−101.84	−72.78	−128.93	−22.07	−129.17	−38.46
Rc&Mc&Mh&Dv	−102.17	−71.53	−127.41	−21.06	−128.12	−35.52
Rc&Mh	−101.41	−76.55	−131.62	−23.97	−126.93	−40.27
Rc&Mh&Dv	−101.65	−72.62	−128.45	−21.67	−128.36	−37.24

Rc, *R. cellulolyticum*, Mc, *M. concilii*, Mh, *M. hungatei*, Dv, *D. vulgaris*.

### Community complexity increases syntrophic dependency and methanogenic activity in *M. concilii*

**Figure 3:**
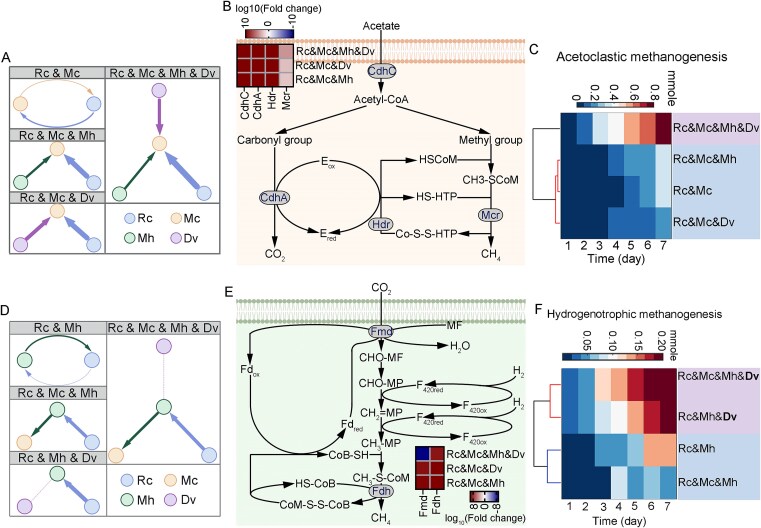
Metabolic activities of *M. concilii* and of *M. hungatei* in synthetic communities. (A) Cross-feeding interactions between *M. concilii* and other species in all synthetic communities. (B) Functional proteins profiling of acetoclastic methanogenesis. (C) Flux of acetoclastic methanogenesis modeled in *M. concilii*. (D) Cross-feeding interactions between *M. hungatei* and other species in all synthetic communities. (E) Functional proteins profiling of hydrogenotrophic methanogenesis. (F) Flux of hydrogenotrophic methanogenesis modeled in *M. hungatei*. Rc: *Ruminiclostridium cellulolyticum,* Mc: *Methanosaeta concilii*, Mh: *Methanospirillum hungatei*, Dv: *Desulfovibrio vulgaris,* CdhC: acetyl-CoA decarboxylase/synthase, CdhA: anaerobic carbon-monoxide dehydrogenase, Hdr: heterodisulfide reductase, Mcr: methyl-coenzyme M reductase, Fmd: formylmethanofuran dehydrogenase, Fdh: formate dehydrogenase.

*Metanosaeta concilii* showed increased dependency on other species as community complexity rose. In tri- and quad-cultures, SMETANA scores were zero when *M. concilii* acted as a donor, indicating no detectable metabolic contribution. Instead, it functioned solely as a receiver, relying on partner-derived metabolites. Compared to the *R. cellulolyticum*–*M. concilii* bi-culture, the SMETANA score between these two species increased by 72.6% in the quad-culture (Table 2 and Fig. 3A), indicating stronger metabolic interdependence with complexity.

Metaproteomic analysis supported this trend. The abundance of four enzymes associated with acetoclastic methanogenesis in *M. concilii*—acetyl-CoA decarboxylase/synthase (CdhC), carbon monoxide dehydrogenase (CdhA), heterodisulfide reductase (Hdr), and methyl-coenzyme M reductase (Mcr)—significantly increased in tri- and quad-cultures compared to the bi-culture (fold change >2.0, *q* < 0.0001) (Fig. 3B). This upregulation likely reflects increased acetate availability, consistent with *R. cellulolyticum* shifting toward hydrogenic acetogenesis in more complex cultures (Fig. 2D). These results suggest *that M. concilii*’s methanogenic activity is primarily substrate-driven, while community complexity may indirectly influence its role by modulating upstream fluxes.

Acetoclastic methanogenesis flux was modeled in all cultures containing *M. concilii* (Fig. 3C). In the bi-culture with *R. cellulolyticum*, flux was 0.32 ± 0.03 mmol. Adding *M. hungatei* had no significant effect (*P* = .87), while *D. vulgaris* reduced flux by 28% (*P* = .005). The quad-culture showed a 153% increase compared to the tri-culture of *R. cellulolyticum*, *M. concilii*, and *M. hungatei* (*P* < .0001), and a 47% increase relative to the combined fluxes of the bi-culture and *R. cellulolyticum*–*M. concilii*–*D. vulgaris* tri-culture (*P* = .006). These trends align with thermodynamic predictions. Although acetoclastic methanogenesis is highly exergonic (ΔG′ ≈ −36 kJ/mol) (Table 3), its flux depends on acetate availability and substrate competition. Reduced flux with *D. vulgaris* likely reflects competition, while the increase in the quad-culture indicates enhanced acetate production and sharing. These findings suggest that *M. concilii*’s methanogenesis is driven by acetate supply and strengthened in complex communities through improved cooperative exchange.

### Reduced metabolic contribution of *M. hungatei* and enhanced methanogenesis

The metabolic contribution of *M. hungatei* decreased as community complexity increased, particularly with *D. vulgaris* and *M. concilii* present (Fig. 3D). In the bi-culture with *R. cellulolyticum*, *M. hungatei* acted as a donor with an SMETANA score of 0.82. Adding *D. vulgaris* to form a tri-culture reduced the donor score to zero. A similar reduction occurred in more complex assemblies. In the tri-culture of *R. cellulolyticum*, *M. hungatei*, and *M. concilii*, the donor score was 2.54, which decreased by 18.3% upon addition of *D. vulgaris* to form the quad-culture. Along with reduced donor contribution, *M. hungatei* showed decreased dependence on community-derived metabolites with greater complexity. As a receiver in the *R. cellulolyticum*–*M. hungatei* bi-culture, its SMETANA score was 4.85, which dropped by 11.5% with *M. concilii* added. Similarly, in the tri-culture of *R. cellulolyticum*, *M. hungatei*, and *D. vulgaris*, the receiver score was 4.59, decreasing to 4.03 when *M. concilii* was included in the quad-culture. These findings suggest that *M. hungatei* becomes metabolically marginalized in higher-order consortia, with both contribution and reliance constrained by more competitive or dominant species like *D. vulgaris* and *M. concilii*.

Two key enzymes in hydrogenotrophic methanogenesis, formylmethanofuran dehydrogenase (Fmd) and formate dehydrogenase (Fdh), were detected across co-cultures (Fig. 3E). Compared to the *R. cellulolyticum*–*M. hungatei* bi-culture, their abundance significantly increased in tri- and quad-cultures (fold change >30, *q* < 0.0001), indicating elevated methanogenesis activity driven by more complex interactions.

*Desulfovibrio vulgaris* significantly enhanced hydrogenotrophic methanogenesis efficiency (Fig. 3F). Over seven days, its presence led to a 78.6% increase in flux (*P* = .01) compared to the *R. cellulolyticum*–*M. hungatei* bi-culture. The flux also rose by 525% in the quad-culture compared to the tri-culture of *R. cellulolyticum*, *M. concilii*, and *M. hungatei* (*P* < .0001). These findings align with thermodynamics, as hydrogenotrophic methanogenesis is strongly exergonic (ΔG′ ≈ −131 kJ/mol) under low H₂ conditions (Table 3). *Desulfovibrio vulgaris* likely promotes hydrogen turnover, maintaining low partial pressures that favor this pathway. This supports efficient electron transfer and explains the enhanced methanogenesis seen in complex communities.

### *Desulfovibrio vulgaris* modulates hydrogen metabolism for facultative syntrophy

**Figure 4:**
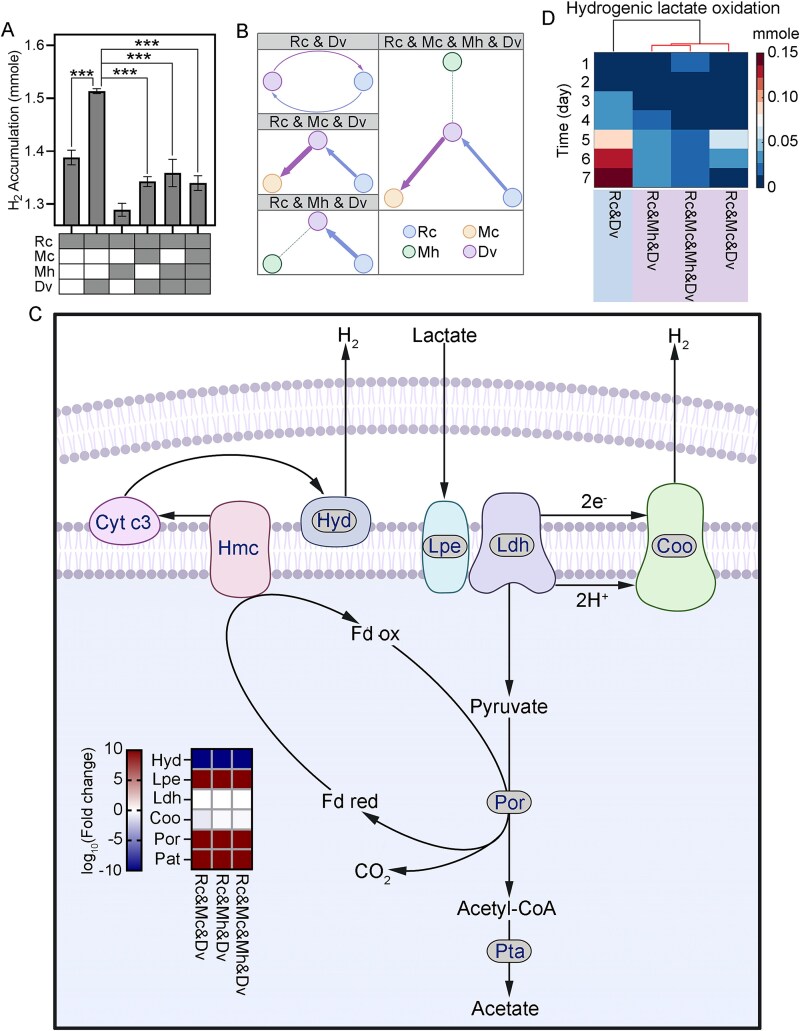
Metabolic activities of *D. vulgaris* in synthetic communities. (A) Comparison of H_2_ accumulation in the cocultures with *D. vulgaris*. Significant differences with *P*-values determined using Student’s *t*-test, and adjusted for the false discovery rate, are marked by ^*^ for *P*-value < 0.05, ^**^ for *P*-value < 0.01, and ^***^ for *P*-value < 0.001. (B) Cross-feeding interactions between *M. hungatei* and other species in all synthetic communities. (C) Functional proteins profiling of hydrogenic lactate oxidation. (D) Flux of hydrogenic lactate oxidation modeled in *D. vulgaris*. Rc: *Ruminiclostridium cellulolyticum,* Mh: *Methanospirillum hungatei,* Mc: *Methanosaeta concilii*, Dv: *Desulfovibrio vulgaris,* Cyt c3: cytochrome C3, Hmc: high-molecular cytochrome, Hyd: hydrogenase, Lpe: L-lactate permease, Ldh: L-lactate dehydrogenase, Coo: membrane-bound Coo hydrogenase, Por: pyruvate:ferredoxin oxidoreductase, Pta: phosphate acetyltransferase.

*Desulfovibrio vulgaris* oxidizes lactate to acetate, CO_2,_ and H_2_ in the absence of sulfate [47]. The accumulation of H₂ was differentially influenced by two methanogens—*M. concilii* and *M. hungatei*. Co-culturing *D. vulgaris* with *M. concilii* resulted in lower H₂ accumulation compared to co-culturing with *M. hungatei* (Fig. 4A). Introducing *M. hungatei* to the bi-culture of *R. cellulolyticum* and *D. vulgaris* led to a 13.2% decrease in H_2_ accumulation (*P*-value = .005), which is comparable to the 13.0% decrease observed in the tri-culture of *R. cellulolyticum*, *D. vulgaris,* and *M. hungatei* compared to the mono-culture (*P*-value = .0008). Adding *M. concilii* to the same bi-culture resulted in a 14.5% reduction in H_2_ accumulation (*P*-value < .0001). However, the subsequent addition of *M. hungatei* to this tri-culture did not lead to significant changes in H_2_ accumulation compared to the tri-culture baseline. Although *M. concilii* does not metabolize hydrogen, metaproteomic analysis showed increased expression of acetoclastic methanogenesis enzymes in multi-species cultures (Fig. 3B), while hydrogenase expression in *R. cellulolyticum* decreased. These results indicate that the presence of *M. concilii* is associated with lower hydrogen accumulation and shifts in fermentative metabolism across the community.

The presence of *M. concilii* or *M. hungatei* affected the metabolic contribution of *D. vulgaris* to community function in contrasting ways—*M. concilii* increased the contribution, while *M. hungatei* decreased it (Fig. 4B). When *D. vulgaris* acted as the metabolic donor, the addition of *M. concilii* to the *R. cellulolyticum*–*D. vulgaris* bi-culture increased its SMETANA score by 114.6%. In contrast, the addition of *M. hungatei* decreased the SMETANA score of *D. vulgaris* to zero. Furthermore, in the tri-culture of *R. cellulolyticum*, *M. concilii,* and *D. vulgaris*, adding *M. hungatei* reduced the SMETANA score of *D. vulgaris* by 21.4%. When *D. vulgaris* acted as receiver, *R. cellulolyticum* was its primary metabolic donor, its SMETANA score increased by 34.0% with *M. concilii* and by 102.4% with *M. hungatei*, compared to bi-culture. However, the combined presence of both *M. concilii* and *M. hungatei* in the quad-culture resulted in only a 42.5% increase, which is less than the additive effect of their individual contributions. This suggests a negative synergistic interaction between *M. concilii* and *M. hungatei* in shaping the metabolic dependency of *D. vulgaris*.

Metaproteomics identified six key enzymes in the hydrogenic lactate oxidation pathway of *D. vulgaris*, L-lactate permease (Lpe), L-lactate dehydrogenase (Ldh), pyruvate:ferredoxin oxidoreductase (Por), phosphate acetyltransferase (Pta), periplasmic [NiFeSe] hydrogenase (Hyd), and membrane-bound Coo hydrogenase (Coo) (Fig. 4C). Compared to the bi-culture of *R. cellulolyticum* and *D. vulgaris*, the abundance of L-lactate permease, pyruvate:ferredoxin oxidoreductase, and phosphate acetyltransferase—enzymes involved in lactate fermentation—significantly increased in tri-cultures and quad-cultures, correlating with decreased lactate accumulation. Conversely, the abundance of periplasmic [NiFeSe] hydrogenase, involved in H₂ production, decreased by up to 92.3-fold (*q*-value < 0.0001) in the tri-culture of *R. cellulolyticum*, *M. concilii*, and *D. vulgaris*, and by 30-fold (*q*-value <0.0001) in the tri-culture of *R. cellulolyticum*, *M. hungatei*, and *D. vulgaris*, as well as in the quad-culture. These findings were supported by flux modeling of hydrogenic lactate oxidation across all community combinations (Fig. 4D). In the bi-culture of *R. cellulolyticum* and *D. vulgaris*, the flux of hydrogenic lactate oxidation was 0.18 ± 0.01 mmol. This flux decreased significantly in the tri-cultures and quad-culture, with an 89.9% reduction in the quad-culture. Although hydrogenic lactate oxidation is exergonic (ΔG′ ≈ −36 kJ/mol), the thermodynamic benefit is maximized under low H₂ conditions (Table 3). The presence of hydrogenotrophic methanogens likely maintains low hydrogen partial pressure, improving the energy yield of lactate oxidation and facilitating interspecies hydrogen transfer. Thus, the observed reduction in H₂ accumulation reflects tighter syntrophic coupling with hydrogen consumers, not suppression of lactate metabolism. This shift enables more efficient energy conservation and contributes to overall metabolic stability in complex microbial communities.

### Microbial interactions from modeling carbon and energy flow

**Figure 5:**
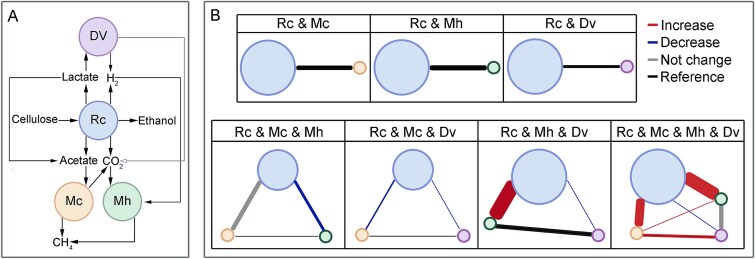
Stoichiometric modeling of synthetic communities. (A) Conceptual metabolic network among four microbial species in the synthetic community. (B) Exchanging fluxes estimating the metabolic interactions between species. The edges represent the exchange flux, and the circles represent the biomass. Rc: *Ruminiclostridium cellulolyticum,* Mh: *Methanospirillum hungatei,* Mc: *Methanosaeta concilii*, Dv: *Desulfovibrio vulgaris.*

The stoichiometric model estimated metabolic exchange fluxes to infer interactions among community members (Fig. 5A). Interactions between *R. cellulolyticum* and *M. concilii* were linked to acetate fluxes, those with *M. hungatei* to H₂ and CO₂, and those with *D. vulgaris* to lactate and H₂. The model also inferred interactions between *M. concilii* and *M. hungatei* via CO₂, *M. concilii* and *D. vulgaris* via acetate, and *M. hungatei* and *D. vulgaris* via lactate and H₂. Variations in metabolic pathways and partner efficiency likely explain community-dependent interaction differences.

Quantitative analysis of the bi-cultures revealed substantial variability in the strength of these metabolic exchanges (Fig. 5B). The strongest interaction occurred between *R. cellulolyticum* and *M. hungatei,* with H_2_ and CO_2_ exchange flux of 0.68 ± 0.003 mmol. This was followed by the acetate exchange flux between *R. cellulolyticum* and *M. concilii*, measured at 0.32 ± 0.03 mmol. The weakest interaction was observed between *R. cellulolyticum* and *D. vulgaris*, with a lactate and H_2_ exchange flux of only 0.18 ± 0.001 mmol. These findings suggest that the strength of metabolic exchange was highly dependent on metabolic complementation.

The nature of microbial relationships—such as cooperation, inhibition, or competition—and their intensity were significantly altered by community complexity, impacting overall metabolic dynamics and efficiency (Fig. 5B). Compared to the bi-culture of *R. cellulolyticum* and *M. hungatei*, the addition of *M. concilii* reduced the H_2_ and CO_2_ exchange flux of H_2_ and CO_2_ by 72.1% (*P*-value < .0001). Conversely, the inclusion of *D. vulgaris* increased the exchange flux by 72.1% (*P*-value = .006). Subsequent addition of *M. concilii* to this tri-culture caused no significant change in the exchange flux (*P*-value = .76), suggesting that *M. concilii* weakened the interaction between *R. cellulolyticum* and *M. hungatei* in the absence of *D. vulgaris*. This pointed to a dynamic equilibrium in the quaternary interaction between *R. cellulolyticum* and *M. hungatei*. In the bi-culture of *R. cellulolyticum* and *M. concilii*, the acetate exchange flux remained unaffected with the addition of *M. hungatei* (*P*-value = .87) but decreased by 31.2% (*P*-value = .004) upon the inclusion of *D. vulgaris*. However, the subsequent addition of *M. hungatei* to this tri-culture led to a 146.9% increase in exchange flux (*P*-value < .0001), indicating that *M. hungatei* mitigated the inhibitory effect of *D. vulgaris* on the interaction between *R. cellulolyticum* and *M. concilii* in the quad-culture. Similar trends were observed for the bi-culture of *R. cellulolyticum* and *D. vulgaris*, where the addition of *M. concilii* reduced the H_2_ and lactate exchange flux by 88.9% (*P-*value < .0001), with a further reduction of 61% (*P*-value = .005) upon subsequent addition of *M. hungatei*. Remarkably, the simultaneous presence of both *M. concilii* and *M. hungatei* in the quad-culture increased the exchange flux by 83.3% (*P*-value < .0001), indicating a synergistic enhancement of microbial interactions, where the inhibitory effects of *M. concilii* and *M. hungatei* on the interaction between *R. cellulolyticum* and *D. vulgaris* were mitigated.

Beyond the common pairwise exchanges, three emergent fluxes were identified in the tri-cultures and the quad-culture. In the tri-cultures, a CO_2_ flux of 0.012 ± 0.001 mmol was observed between *M. concilii* and *M. hungatei,* while an acetate flux of 0.005 ± 0.001 mmol was detected between *M. concilii* and *D. vulgaris*. Both fluxes increased by 75%–88% (*P*-value < .0001) in the quad-culture. Meanwhile, an H_2_ flux of 0.07 ± 0.02 mmol between *M. hungatei* and *D. vulgaris* remained unchanged in the quad-culture (*P*-value = .78). These observations underscored the complexity and dynamic nature of metabolic interactions in multi-species communities, characterized by emergent fluxes and altered interactions.

These fluxes aligned with thermodynamic favorability. Hydrogenotrophic methanogenesis (ΔG′ ≈ −131 kJ/mol) likely drove the strong *R. cellulolyticum*–*M. hungatei* interaction. The acetate flux with *M. concilii* reflected favorable acetoclastic methanogenesis (ΔG′ ≈ −36 kJ/mol). Weaker exchanges with *D. vulgaris* likely result from tighter energy margins in lactate fermentation (ΔG′ ≈ −32 kJ/mol) and substrate competition (Table 3). Overall, the thermodynamic context helps explain cooperative dynamics in increasingly complex microbial communities.

## Discussion

Anaerobic microbial interactions in carbon and electron transfer have been well characterized in natural and simplified lab systems. Prior studies defined key functions of hydrogenotrophic and acetoclastic methanogens, sulfate reducers, and cellulolytic fermenters, highlighting cross-feeding’s role in methane production and redox balance. However, most insights stem from pairwise cultures or descriptive omics, lacking resolution on how interaction networks change in complex communities [24, 48–50]. In this study, we address this limitation by using a fully combinatorial four-species synthetic system to explore how complexity reshapes cooperation and competition during cellulose degradation to CH₄. The model organisms used have defined metabolic roles, genomic resources, and ecological relevance, providing a tractable, interpretable platform. Unlike studies testing hypotheses or reporting correlations, our approach integrates SMETANA modeling, stoichiometric flux analysis, and proteogenomics to resolve how metabolic roles shift with community configuration. The aim was not pathway discovery but to build a quantitative foundation for understanding context-dependent syntrophic dependencies.

Our results demonstrate that community complexity alters both the magnitude and structure of metabolic interactions in nonlinear, context-specific ways. Tri-cultures often had stronger cooperation than bi-cultures, with increased MIP and reduced MRO. While the four-species community had lower MIP than some tri-cultures, this result reflects a single configuration and cannot be generalized. Rather than species richness suppressing cooperation, our data suggest that richness reshapes interaction networks based on partner compatibility. These results align with recent studies showing that interaction strength is driven more by functional traits and network topology than community size [51–53].

*Ruminiclostridium cellulolyticum* consistently functioned as the central metabolic donor. In more complex configurations, it showed regulatory changes that supported community-level syntrophy. For example, it decreased hydrogen and cellulase production while shifting carbon flux toward hydrogenic acetogenesis. Thermodynamically, this pathway is only mildly favorable in monoculture (ΔG′ ≈ −7.5 kJ/mol) but becomes more favorable under syntrophic conditions (ΔG′ ≈ −29 kJ/mol in the quad-culture). These adjustments minimized product accumulation, facilitated electron flow, and prioritized community-level energy balance over individual energy yield, consistent with other syntrophic fermenters [54, 55].

Methanogens showed divergent responses. *Methanosaeta concilii* became a fully dependent receiver in tri- and quad-cultures while increasing its methanogenic output. This was marked by elevated expression of acetoclastic pathway enzymes and increased flux, especially when *D. vulgaris* was present, likely enhancing acetate availability through cross-feeding. Acetoclastic methanogenesis, being highly exergonic (ΔG′ ≈ −36 kJ/mol), serves as an efficient sink for carbon and electrons under cooperative conditions (Table 3) [56–58]. In contrast, *M. hungatei* showed decreased donor and receiver roles with increasing complexity but displayed substantially enhanced hydrogenotrophic methanogenesis in the quad-culture. This decoupling of exchange roles and functional output mirrors observations in facultative syntrophs, where metabolic roles persist even with diminished network centrality [32]. Given that hydrogenotrophic methanogenesis is highly favorable (ΔG′ ≈ −131 kJ/mol), it provides a robust electron sink for the community (Table 3).

*Desulfovibrio vulgaris* emerged as a key modulator of community metabolism. While its donor role varied by species context, its presence consistently improved both acetoclastic and hydrogenotrophic methanogeneses. Proteomic and flux data showed increased lactate oxidation alongside reduced hydrogenase expression and hydrogen flux. This indicates that hydrogen was still produced but consumed rapidly by methanogens such as *M. hungatei*, maintaining low partial pressures and thermodynamic favorability for lactate oxidation (ΔG′ ≈ −26 kJ/mol) (Table 3) [59, 60]. Rather than eliminating hydrogen production, *D. vulgaris* likely benefits from interspecies hydrogen transfer, using methanogens as efficient electron sinks. This redox balance improves energy efficiency and supports metabolic stability in complex communities*.*

While SMETANA effectively predicted cooperation and competition trends, combining it with proteomic and flux data yielded deeper insight. For instance, the predicted reduction in competition in the quad-culture aligned with proteomic evidence of niche specialization and reduced fermentation redundancy. This multi-layered approach supports systems biology perspectives that emphasize integrating models with omics to uncover emergent community properties beyond what sequence data alone can reveal [61, 62].

It is important to emphasize that these findings derive from a defined four-species system. Although the taxonomic composition is not novel, the full combinatorial design and simplicity allowed precise mapping of metabolic interactions, carbon flows, and species-specific contributions. This framework makes it possible to isolate and study higher-order interactions that are difficult to access in natural microbiomes, offering a foundation for hypothesis-driven analysis of community metabolic dynamics [63].

In summary, this study demonstrates that microbial cooperation and competition are shaped not merely by species richness but by context-dependent metabolic compatibility and partner-specific interactions. By leveraging a fully combinatorial and mechanistically resolved synthetic system, we show that microbial communities dynamically reorganize their metabolic networks in response to changing community context, producing emergent behaviors that are not predictable from pairwise associations or genomic potential alone. These findings advance beyond previous studies by providing a quantitative framework that links species identity, flux distribution, and functional outcomes. This approach offers a foundation for predictive modeling of microbial ecosystems and informs the rational design of stable consortia for applications in bioconversion, waste treatment, and synthetic ecology.

## Data Availability

The proteomics datasets generated during the current study are available in ProteomeXchange Consortium via the PRIDE (Proteomics Identification Database) partner repository with the dataset identifier PXD056517.

## References

[1] Coyte KZ, Schluter J, Foster KR. The ecology of the microbiome: networks, competition, and stability. *Science* 2015;350:663–6. 10.1126/science.aad2602 26542567

[2] Lin Q, Li L, De Vrieze J et al. Functional conservation of microbial communities determines composition predictability in anaerobic digestion. *ISME J* 2023;17:1920–30. 10.1038/s41396-023-01505-x 37666974 PMC10579369

[3] Coban O, De Deyn GB, van der Ploeg M. Soil microbiota as game-changers in restoration of degraded lands. *Science* 2022;375:abe0725. 10.1126/science.abe0725 35239372

[4] Sobral M, Silvius KM, Overman H et al. Mammal diversity influences the carbon cycle through trophic interactions in the amazon. *Nat Ecol Evol* 2017;1:1670–6. 10.1038/s41559-017-0334-0 28993614

[5] Lee H, Bloxham B, Gore J. Resource competition can explain simplicity in microbial community assembly. *Proc Natl Acad Sci* 2023;120:e2212113120. 37603734 10.1073/pnas.2212113120 PMC10469513

[6] Gralka M, Szabo R, Stocker R et al. Trophic interactions and the drivers of microbial community assembly. *Curr Biol* 2020;30:R1176–88. 33022263 10.1016/j.cub.2020.08.007

[7] Weber KA, Achenbach LA, Coates JD. Microorganisms pumping iron: anaerobic microbial iron oxidation and reduction. *Nat Rev Microbiol* 2006;4:752–64. 10.1038/nrmicro1490 16980937

[8] Candry P, Abrahamson B, Stahl DA et al. Microbially mediated climate feedbacks from wetland ecosystems. *Glob Chang Biol* 2023;29:5169–83. 37386740 10.1111/gcb.16850

[9] van den Berg NI, Machado D, Santos S et al. Ecological modelling approaches for predicting emergent properties in microbial communities. *Nat Ecol Evol* 2022;6:855–65. 35577982 10.1038/s41559-022-01746-7 PMC7613029

[10] Gralka M, Pollak S, Cordero OX. Genome content predicts the carbon catabolic preferences of heterotrophic bacteria. *Nat Microbiol* 2023;8:1799–808. 37653010 10.1038/s41564-023-01458-z

[11] Dal Bello M, Lee H, Goyal A et al. Resource–diversity relationships in bacterial communities reflect the network structure of microbial metabolism. *Nat Ecol Evol* 2021;5:1424–34. 34413507 10.1038/s41559-021-01535-8

[12] Kost C, Patil KR, Friedman J et al. Metabolic exchanges are ubiquitous in natural microbial communities. *Nat Microbiol* 2023;8:2244–52. 37996708 10.1038/s41564-023-01511-x

[13] Friedman J, Higgins LM, Gore J. Community structure follows simple assembly rules in microbial microcosms. *Nat Ecol Evol* 2017;1:0109. 10.1038/s41559-017-0109 28812687

[14] Hu J, Amor DR, Barbier M et al. Emergent phases of ecological diversity and dynamics mapped in microcosms. *Science* 2022;378:85–9. 36201585 10.1126/science.abm7841

[15] Walker CB, Redding-Johanson AM, Baidoo EE et al. Functional responses of methanogenic archaea to syntrophic growth. *ISME J* 2012;6:2045–55. 22739494 10.1038/ismej.2012.60 PMC3475374

[16] Fink MM . Examining the Impact of Enterococcus Faecalis on Pseudomonas Aeruginosa Metabolism and Behavior. University of Notre Dame ProQuest Dissertations & Theses, 2024.

[17] Culp EJ, Goodman AL. Cross-feeding in the gut microbiome: ecology and mechanisms. *Cell Host Microbe* 2023;31:485–99. 37054671 10.1016/j.chom.2023.03.016 PMC10125260

[18] Holbrook-Smith D, Trouillon J, Sauer U. Metabolomics and microbial metabolism: toward a systematic understanding. *Annu Rev Biophys* 2023;**53**:41–64. 10.1146/annurev-biophys-030722-021957 38109374

[19] Blair EM, Dickson KL, O’Malley MA. Microbial communities and their enzymes facilitate degradation of recalcitrant polymers in anaerobic digestion. *Curr Opin Microbiol* 2021;64:100–8. 34700124 10.1016/j.mib.2021.09.008

[20] Bharti R, Grimm DG. Current challenges and best-practice protocols for microbiome analysis. *Brief Bioinform* 2021;22:178–93. 31848574 10.1093/bib/bbz155 PMC7820839

[21] Jansson JK, Hofmockel KS. The soil microbiome—from metagenomics to metaphenomics. *Curr Opin Microbiol* 2018;43:162–8. 29454931 10.1016/j.mib.2018.01.013

[22] Moreira ZPM, Chen MY, Ortuno DLY et al. Engineering plant microbiomes by integrating eco-evolutionary principles into current strategies. *Curr Opin Plant Biol* 2023;71:102316. 36442442 10.1016/j.pbi.2022.102316

[23] Franzosa EA, Hsu T, Sirota-Madi A et al. Sequencing and beyond: integrating molecular'omics' for microbial community profiling. *Nat Rev Microbiol* 2015;13:360–72. 25915636 10.1038/nrmicro3451 PMC4800835

[24] Wang D, Hunt KA, Candry P et al. Cross-feedings, competition, and positive and negative synergies in a four-species synthetic community for anaerobic degradation of cellulose to methane. *MBio* 2023;14:e03189–22. 36847519 10.1128/mbio.03189-22 PMC10128006

[25] Wang J, Li X, Jin H et al. Co-driven electron and carbon flux fuels synergistic microbial reductive dechlorination. *Microbiome* 2024;12:154. 39160636 10.1186/s40168-024-01869-y PMC11334346

[26] Li Y, Xue Y, Roy Chowdhury T et al. Genomic insights into redox-driven microbial processes for carbon decomposition in thawing arctic soils and permafrost. *MSphere* 2024;9:e00259–24. 38860762 10.1128/msphere.00259-24 PMC11288003

[27] Wang F, Zhao C, Shi X et al. Warning the environmental risks of emerging contaminants on low-carbon sludge anaerobic digestion treatment. *Curr Opin Environ Sci Health* 2025;43:100592.

[28] Kato S, Haruta S, Cui ZJ et al. Stable coexistence of five bacterial strains as a cellulose-degrading community. *Appl Environ Microbiol* 2005;71:7099–106. 16269746 10.1128/AEM.71.11.7099-7106.2005 PMC1287685

[29] Yang Y . Emerging patterns of microbial functional traits. *Trends Microbiol* 2021;29:874–82. 34030967 10.1016/j.tim.2021.04.004

[30] Goldschmidt F, Caduff L, Johnson DR. Causes and consequences of pattern diversification in a spatially self-organizing microbial community. *ISME J* 2021;15:2415–26. 33664433 10.1038/s41396-021-00942-w PMC8319339

[31] Sahraeian SME, Yoon B-J. Smetana: accurate and scalable algorithm for probabilistic alignment of large-scale biological networks. *PLoS One* 2013;8:e67995. 23874484 10.1371/journal.pone.0067995 PMC3710069

[32] Zelezniak A, Andrejev S, Ponomarova O et al. Metabolic dependencies drive species co-occurrence in diverse microbial communities. *Proc Natl Acad Sci* 2015;112:6449–54. 25941371 10.1073/pnas.1421834112 PMC4443341

[33] Machado D, Andrejev S, Tramontano M et al. Fast automated reconstruction of genome-scale metabolic models for microbial species and communities. *Nucleic Acids Res* 2018;46:7542–53. 30192979 10.1093/nar/gky537 PMC6125623

[34] Li Z, Yao Q, Guo X et al. Genome-resolved proteomic stable isotope probing of soil microbial communities using 13co2 and 13c-methanol. *Front Microbiol* 2019;10:2706. 31866955 10.3389/fmicb.2019.02706 PMC6908837

[35] Yao Q, Li Z, Song Y et al. Community proteogenomics reveals the systemic impact of phosphorus availability on microbial functions in tropical soil. *Nat Ecol Evol* 2018;2:499–509. 29358607 10.1038/s41559-017-0463-5

[36] Walker JM . The bicinchoninic acid (bca) assay for protein quantitation. *The protein protocols handbook* 2009; pp 11–15. 10.1385/0-89603-268-X:5 7951748

[37] Casey TM, Khan JM, Bringans SD et al. Analysis of reproducibility of proteome coverage and quantitation using isobaric mass tags (itraq and tmt). *J Proteome Res* 2017;16:384–92. 28152591 10.1021/acs.jproteome.5b01154

[38] Spivak M, Weston J, Bottou L et al. Improvements to the percolator algorithm for peptide identification from shotgun proteomics data sets. *J Proteome Res* 2009;8:3737–45. 19385687 10.1021/pr801109k PMC2710313

[39] Griffin NM, Yu J, Long F et al. Label-free, normalized quantification of complex mass spectrometry data for proteomic analysis. *Nat Biotechnol* 2010;28:83–9. 20010810 10.1038/nbt.1592 PMC2805705

[40] Vogel C, Marcotte EM. Calculating absolute and relative protein abundance from mass spectrometry-based protein expression data. *Nat Protoc* 2008;3:1444–51. 18772871 10.1038/nport.2008.132

[41] Matzke MM, Brown JN, Gritsenko MA et al. A comparative analysis of computational approaches to relative protein quantification using peptide peak intensities in label-free lc-ms proteomics experiments. *Proteomics* 2013;13:493–503. 23019139 10.1002/pmic.201200269 PMC3775642

[42] Kanehisa M, Furumichi M, Sato Y et al. Kegg: biological systems database as a model of the real world. *Nucleic Acids Res* 2024;**53**:672–677. 10.1093/nar/gkae909 PMC11701520 39417505

[43] Karp PD, Riley M, Paley SM et al. The metacyc database. *Nucleic Acids Res* 2002;30:59–61. 11752254 10.1093/nar/30.1.59 PMC99148

[44] Desvaux M, Guedon E, Petitdemange H. Cellulose catabolism by clostridium cellulolyticum growing in batch culture on defined medium. *Appl Environ Microbiol* 2000;66:2461–70. 10831425 10.1128/aem.66.6.2461-2470.2000 PMC110559

[45] Gupta R, Gupta N, Gupta R et al. Glycolysis and gluconeogenesis. *Fundamentals of bacterial physiology and metabolism,* Springer, 2021;267–87.

[46] Love M, Anders S, Huber W. Differential analysis of count data–the deseq2 package. *Genome Biol* 2014;15:10.1186.

[47] Noguera DR, Brusseau GA, Rittmann BE et al. A unified model describing the role of hydrogen in the growth of desulfovibrio vulgaris under different environmental conditions. *Biotechnol Bioeng* 1998;59:732–46. 10099394 10.1002/(sici)1097-0290(19980920)59:6<732::aid-bit10>3.0.co;2-7

[48] Wang D, Candry P, Hunt KA et al. Metaproteomics-informed stoichiometric modeling reveals the responses of wetland microbial communities to oxygen and sulfate exposure. *NPJ Biofilms Microbiomes* 2024;10:55. 38961111 10.1038/s41522-024-00525-5 PMC11222425

[49] McInerney MJ, Sieber JR, Gunsalus RP. Syntrophy in anaerobic global carbon cycles. *Curr Opin Biotechnol* 2009;20:623–32. 19897353 10.1016/j.copbio.2009.10.001 PMC2790021

[50] Morris BE, Henneberger R, Huber H et al. Microbial syntrophy: interaction for the common good. *FEMS Microbiol Rev* 2013;37:384–406. 23480449 10.1111/1574-6976.12019

[51] Pacheco AR, Osborne ML, Segrè D. Non-additive microbial community responses to environmental complexity. *Nat Commun* 2021;12:2365. 33888697 10.1038/s41467-021-22426-3 PMC8062479

[52] Srinivasan S, Jnana A, Murali TS. Modeling microbial community networks: methods and tools for studying microbial interactions. *Microb Ecol* 2024;87:56. 38587642 10.1007/s00248-024-02370-7 PMC11001700

[53] Berrios L, Venturini AM, Ansell TB et al. Co-inoculations of bacteria and mycorrhizal fungi often drive additive plant growth responses. *ISME Commun* 2024;4:ycae104. 39188310 10.1093/ismeco/ycae104 PMC11346365

[54] Strassfeld DA, Chen C-Y, Park HS et al. Hydrogen-bond-acceptor ligands enable distal c (sp 3)–h arylation of free alcohols. *Nature* 2023;622:80–6. 37674074 10.1038/s41586-023-06485-8 PMC11139439

[55] Saykally RJ, Blake GA. Molecular interactions and hydrogen bond tunneling dynamics: some new perspectives. *Science* 1993;259:1570–5. 17733020 10.1126/science.259.5101.1570

[56] Liu Y, Whitman WB. Metabolic, phylogenetic, and ecological diversity of the methanogenic archaea. *Ann N Y Acad Sci* 2008;1125:171–89. 18378594 10.1196/annals.1419.019

[57] Huang Y, Igarashi K, Liu L et al. Methanol transfer supports metabolic syntrophy between bacteria and archaea. *Nature* 2025;**639**:190–195. 10.1038/s41586-024-08491-w 39880954

[58] Freude C, Blaser M. Carbon isotope fractionation during catabolism and anabolism in acetogenic bacteria growing on different substrates. *Appl Environ Microbiol* 2016;82:2728–37. 26921422 10.1128/AEM.03502-15 PMC4836411

[59] Stams AJ, Plugge CM. Electron transfer in syntrophic communities of anaerobic bacteria and archaea. *Nat Rev Microbiol* 2009;7:568–77. 19609258 10.1038/nrmicro2166

[60] Sieber JR, McInerney MJ, Gunsalus RP. Genomic insights into syntrophy: the paradigm for anaerobic metabolic cooperation. *Ann Rev Microbiol* 2012;66:429–52. 22803797 10.1146/annurev-micro-090110-102844

[61] Machado D, Maistrenko OM, Andrejev S et al. Polarization of microbial communities between competitive and cooperative metabolism. *Nat Ecol Evol* 2021;5:195–203. 33398106 10.1038/s41559-020-01353-4 PMC7610595

[62] Long C, Deng J, Nguyen J et al. Structured community transitions explain the switching capacity of microbial systems. *Proc Natl Acad Sci* 2024;121:e2312521121. 38285940 10.1073/pnas.2312521121 PMC10861894

[63] Widder S, Allen RJ, Pfeiffer T et al. Challenges in microbial ecology: building predictive understanding of community function and dynamics. *ISME J* 2016;10:2557–68. 27022995 10.1038/ismej.2016.45 PMC5113837

